# Efficacy and safety of acupoint injection therapy for allergic rhinitis: A protocol for systematic review and meta-analysis

**DOI:** 10.1097/MD.0000000000022737

**Published:** 2020-10-16

**Authors:** Zhenfeng Chen, Shasha Xing, Yingzhuan Huang, Qifeng Zeng, Donghan Xu, Qiumei He, Huangguan Qin, Xiaofan Luo, Renfeng Li

**Affiliations:** a Guangxi university of Traditional Chinese Medicine; b Ruikang Hospital affiliated to Guangxi University of Chinese Medicine, Nanning, Guangxi Province; c Macau University of Science and Technology, College of Traditional Chinese Medicine Macau; d The first Affiliated Hospital of Guangxi University of Chinese Medicine, Nanning, Guangxi Province, China.

**Keywords:** acupoint injection, allergic rhinitis, protocol, systematic review

## Abstract

**Background:**

Allergic rhinitis (AR), characterized by nasal itching, sneezing, and congestion, is a common disorder of nose. In the United States, AR affects 10% to 20% of adults. The negative impact of the high prevalence of AR has caused a great economic burdens worldwide. Modern Western Medicine mainly treats AR with antihistamine drugs, glucocorticoids, allergic immunotherapy (AIT), but it seriously affects patients compliance because of its long course of treatment, high medical costs and side effect. And now, as an important mean of treating AR, acupoint injection has been widely used in clinics, and has achieved significant efficacy.

**Methods and analysis:**

The following databases will be searched for relevant information before July 2020: PubMed, Embase, Cochrane Library, Web of Science, and CNKI. Major results: scores of Rhinitis Quality of Life (RQLQ), Rhinitis Total Symptom Scores (RTSS). Secondary results: levels of antigen-specific serum immunoglobulin E (IgE), total effective rate, adverse event. Data will be collected independently by 2 researchers, and the risk of bias in meta-analysis will be evaluated according to “Cochrane Handbook for Systematic Reviews of Interventions”. All data analysis will be conducted using Review Manager V.5.3. and Stata V.12.0.

**Results:**

The curative effect and safety of acupoint injection treatment for AR patients will be evaluated systematically.

**Conclusion:**

The systematic review of this study will summarize the currently published evidence of acupoint injection treatment for AR to further guide its promotion and application.

**Ethics and dissemination:**

The private information from individuals will not be published. This systematic review also will not involve endangering participant rights. Ethical approval is not required. The results may be published in a peer-reviewed journal or disseminated in relevant conferences.

Open Science Framework (OSF) registration number: https://osf.io/fa9dq.

## 1 Introduction

Allergic rhinitis (AR) is a non-infectious inflammatory disease of nasal mucosa mediated by IgE after atopic individuals are exposed to allergens.^[1]^ The main clinical manifestations are nasal itching, stuffy nose, sneezing, watery nose, congestion, and so on. AR affects 10% to 20% of adults in the United States.^[2]^ Due to the easy relapse of AR and the delay in the course of the disease, some AR patients are often complicated with asthma, nasal polyps, and rhinosinusitis.^[3]^ AR can cause adverse effects impacting patients quality of life (including impairment of physical and social functioning, sleep disorders, daytime sleepiness, learning and memory impairment, and psychology disorders).^[4–6]^ The negative impact of the high prevalence of AR has caused a great economic burdens worldwide.

Therefore, it is very necessary to actively treat AR and improve the quality of life of patients. Modern western medicine treatment of AR mainly antihistamine drugs, hormone therapy, allergy immunotherapy (AIT) and surgical treatment. Although drug treatment has a good short-term efficacy, it is easy to relapse after drug withdrawal.

AS thus, particularly, it is urgent and important to explore an alternative therapy. In recent years, acupoint injection has been widely used in the treatment of AR, and played a good role in improving symptoms and quality of life. Moreover, it is one of the methods of integrated traditional Chinese and western medicine in the treatment of allergic rhinitis, which as a comprehensive therapy, mainly based on the combination of acupoints, drugs and meridians. By injecting a small amount of liquid drugs into specific acupoints and producing stimulation, it can enhance and adjust the immune function of the body, and finally achieve the purpose of curing the disease.^[7]^ Hence, the aim of this study was to evaluate the efficacy and safety of acupoint injection in the treatment of AR by meta-analysis.

## 2 Objectives

In a randomized controlled trial (RCT), the efficacy and side effects of acupoint injection in treating AR have been evaluated systematically. We expect to provide reference for AR treatment in the field of acupoint injection.

## 3 Methods

### 3.1 Study registration

The protocol of the systematic review has been registered.

Registration: OSF Preregisration.2020, Sep.8. osf.io/fa9dq. This systematic review protocol will be conducted and reported strictly according to Preferred Reporting Items for Systematic Reviews and Meta-Analyses (PRISMA)^[8]^ statement guidelines, and the important protocol amendments will be documented in the full review.

### 3.2 Inclusion and exclusion criteria for study selection

#### 3.2.1 Inclusion criteria

Inclusion criteria are all randomized controlled trials (RCTs), which main treatment of AR is acupoint injection. The language of the trials to be included only Chinese or English.

#### 3.2.2 Exclusion criteria

Following studies will be excluded:

1. Repeated publications
2. Review of literature and cases
3. Animal studies
4. Incomplete literature
5. Non-randomized controlled trials

### 3.3 Types of participants

We will include RCTs of participants of 18 years or older, of any sex, race/ethnicity, and the patients are in accordance with diagnostic criteria of AR established by the American Academy of Otolaryngology or Chinese Medical Association of Otorhinolaryngology. We will exclude patients who are also participating in clinical trials for other drugs or treatments; patients with mental or legal disabilities should not be treated with this therapy; pregnant or lactating women, mental patients, etc.; patients with severe diseases of the heart, brain, liver, kidney or hematopoietic system.

### 3.4 Interventions and controls

Interventions included treatment with acupoint injection. The control group only received conventional western medicine treatment. The routine treatment of each RCT may not be identical, but the use of acupoint injection is the only difference between intervention and control.

### 3.5 Type of outcome measures

#### 3.5.1 Main outcomes

1. Rhinitis Quality of Life (RQLQ);
2. Rhinitis Total Symptom Scores (RTSS);

#### 3.5.2 Additional outcomes

1. antigen-specific serum IgE;
2. total effective rate;
3. adverse events.

### 3.6 Search methods

#### 3.6.1 Search resources

**Figure 1:**
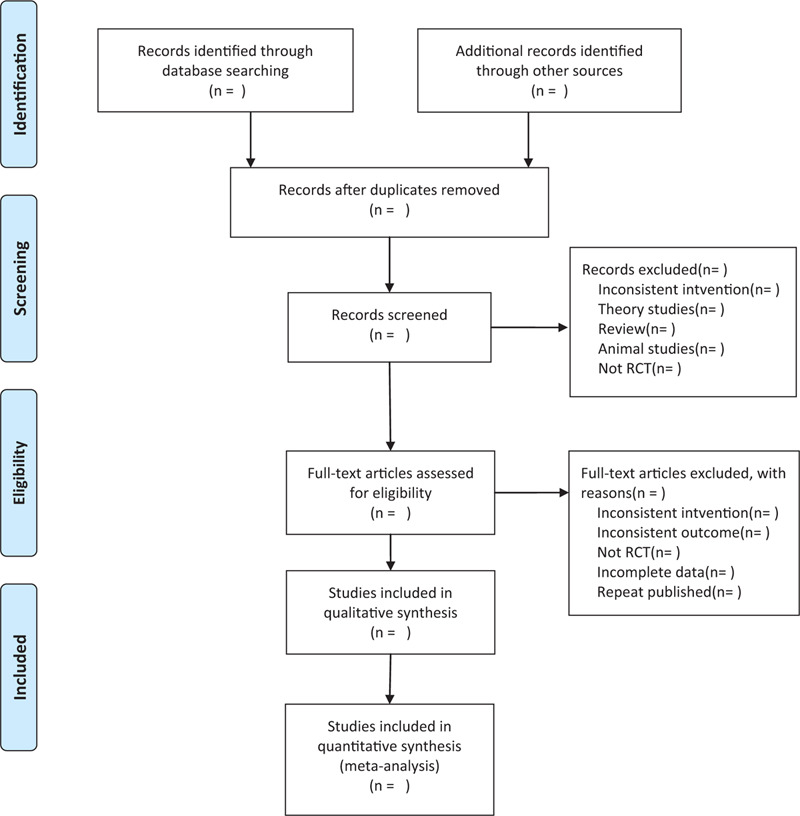
The research flowchart. This figure shows the identification, screening, eligibility and included when we searching articles.

This review will include the following electronic databases from their inception to Aug 2020: Electronic database includes PubMed, Embase, Cochrane Library, Web of Science, CNKI (Fig. 1). The research flowchart. We will not apply any language restrictions.

#### 3.6.2 Search strategies

The following MeSH terms and their combinations will be searched:

- AR;
- RCT OR RCTs;
- acupoint injection OR point injection OR acupuncture point injection.

The search strategy for PubMed is shown in (Table 1). Other electronic data bases will be used the same strategy.

**Table 1:**
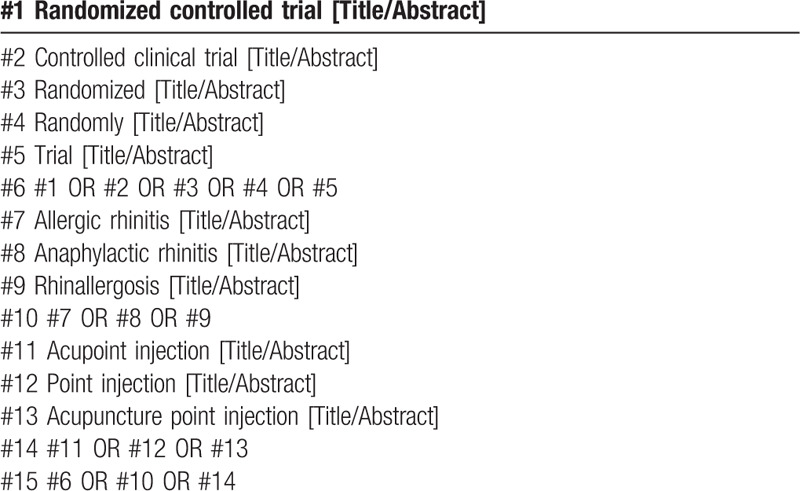
Search strategy in PubMed database.

### 3.7 Data collection and analysis

#### 3.7.1 Studies selection

Two researchers (ZC and SX) will independently assess abstracts and titles of studies identified by literature search. Duplicates will be omitted using EndNote software (V.X9.0). Secondly, we will download full text of the relevant studies for further selection according to the inclusion criteria. If there is any different opinion, 2 researchers will discuss and reach an agreement. If a consensus could not be reached, there will be a third researcher (RL) who make the final decision. The details of selection process will be displayed in the PRISMA flow chart.

#### 3.7.2 Assessment of risk of bias

The assessment of risk of bias will be carried out by 2 independent reviewers (ZC and YH), using the Cochrane Collaboration's “Risk of bias” tool. Study bias will be conducted as either: “unclear,” “low,” or “high” risk for the following criteria: random sequence generation, allocation concealment, blinding, incomplete data, selective outcome reporting, and other bias. The assessment of the bias has caused controversy, there is a need for discussion with a third reviewer (RL). The graphic representations of potential bias within and across studies using Rev Man V.5.3.5.

#### 3.7.3 Measures of treatment effect

Statistical analyses will be conducted using the risk ratio with 95% confidence intervals (CIs). Odds ratio (OR) and relative risk (RR) are commonly used for dichotomous outcomes data. For continuous outcomes, the weighted mean difference (WMD) or the standard mean difference (SMD) will be analyzed.

#### 3.7.4 Unit of analysis issues

The unit of analysis will be the individual participant.

#### 3.7.5 Dealing with missing data

Among the results of several studies with insufficient data or missing data, the corresponding author will be contacted to complement the contents. If the corresponding author cannot be contacted, the data alone will be conducted.

#### 3.7.6 Assessment of heterogeneity

The assessment of heterogeneity will be conducted by Review Manager (V.5.3.5). Chi-squared test and *I*^2^value of the forest, plot will be calculated to assess heterogeneity, according to the Cochrane Handbook. The *I*^2^value is classified into 4 levels: little or no heterogeneity (0%–40%), moderate heterogeneity (30%–60%), substantial heterogeneity (50%–90%), and considerable heterogeneity (75%–100%).

#### 3.7.7 Assessment of reporting biases

If the numbers of available studies are sufficient, funnel plots will be assessed reporting biases.

#### 3.7.8 Data synthesis

Review Manager (V.5.3.5) will be used to analyze. The test indicated little or no heterogeneity; a fixed effect model will be used for data. The random effect model will be adopted when there is considerable heterogeneity (*I*^2^ ≥ 50%). If there is considerable variation in results (*I*^2^ ≥ 75%), the meta-analysis will not be performed. The narrative and qualitative summary will be available.

#### 3.7.9 Subgroup analysis and investigation of heterogeneity

Subgroup analysis will be conducted to assess heterogeneity. The different types of drugs of acupoint injection (antihistamine, immunopotentiator, glucocorticoid, vitamin B12, Chinese Medicine Preparation) may be affected heterogeneity.

#### 3.7.10 Sensitivity analysis

Sensitivity analysis will be used to assess the robustness of the results. It is possible to determine according to methodological quality, sample size, and analysis-related issues. The studies that follow a sequence will be removed from all the inclusion reviews. The Chi-Squared test and *I*^2^ value will be used to quantify statistical heterogeneity.

#### 3.7.11 Summary of evidence

The assessment of evidence for all outcomes will be summarized using the Grading of Recommendations Assessment, Development and Evaluation (GRADE) approach. The quality of evidence will be rated as high, moderate, low, and very low quality.

## 4 Discussion

Obviously, allergic rhinitis has become a public health problem that needs to be paid attention to. AR belongs to type I hypersensitivity that is mainly mediated by IgE, during which a variety of immunoreactive cells (Th1, Th2), inflammatory mediators (including interleukin IL-4, IL-5) and inflammatory cells (such as T cells, mast cells, eosinophils and basophils) are activated.^[9–10]^ Western medicine prefer to relieve symptoms of AR, and most patients are treated with antihistamine drugs, glucocorticoids or AIT. Although its fast effect in improving symptoms, there are side effects, immune therapy for a long time and high medical bills, seriously affect the patients compliance, also prone to adverse reactions.

Study proves that acupuncture can generate a curative effect on this disease by restoring Th1/Th balance, decreasing IgE levels, reducing nasal mucosal inflammatory cell infiltration. Acupoint injection therapy is based on the basic theory of acupuncture, and under the guidance of meridian theory, it combines meridians, acupoints and drug effects. Through the action of meridians and acupoints, the drug effect can be magnified, so only a few drugs can produce a good curative effect. The mechanism of acupoint injection therapy is to ameliorate the pathological conditions and regulate the function of the body through the synergistic effect of acupuncture and drugs.^[11–12]^ The effects of drugs include not only differentiation of traditional Chinese medicine, but also modern pharmacology. It has the advantages of short course of treatment, excellent therapeutic effect, low cost, and good security, which has been widely used in the clinical treatment of various diseases. The report show that acupoint injection can significantly improve the clinical symptoms and quality of life of AR patients.^[13]^ In addition, acupoint injection can relieve the nasal mucosa inflammation by reduce the levels of H1R and H4R proteins and decreasing the gathering of EOS in the nasal mucosa. Through many clinical samples, it is fully realized that acupoint injection has its advantages in treating AR.^[14–15]^

In short, this systematic review and meta-analysis can help identify the potential value of acupoint injection in the treatment of AR and improving patients QOL. Furthermore, this study will provide a basis for patient selection treatment options in future.

## Author contributions

**Conceptualization:** Zhenfeng Chen, Shasha Xing.

**Data curation:** Shasha Xing, Yingzhuan Huang, Renfeng Li.

**Formal analysis:** Zhenfeng Chen, Donghan Xu, Qiumei He.

**Funding acquisition:** Renfeng Li.

**Investigation:** Shasha Xing, Yingzhuan Huang.

**Project administration:** Renfeng Li.

**Quality assessment:** Renfeng Li, Zhenfeng Chen.

**Software:** Yingzhuan Huang, Shasha Xing, Xiaofan Luo.

**Supervision:** Zhenfeng Chen.

**Validation:** Zhenfeng Chen, Lizhen Wang.

**Writing – original draft:** Zhenfeng Chen, Shasha Xing, Yingzhuan Huang.

**Writing – review & editing:** Shasha Xing, Renfeng Li.
